# Morphology, Glycan Pattern, Heat Shock Proteins, and Sex Steroid Receptors Expression in the Tubal Fimbria Epithelium of the Baboon *Papio hamadryas* during the Menstrual Cycle

**DOI:** 10.3390/ani14162321

**Published:** 2024-08-11

**Authors:** Salvatore Desantis, Mario Cinone, Luca Lacitignola, Pietro Laricchiuta, Roberta Rossi, Antonio Ciro Guaricci, Leonardo Resta, Maria Albrizio

**Affiliations:** 1 Department of Precision and Regenerative Medicine and Jonian Area (DiMePRe-J), University of Bari Aldo Moro, 70124 Bari, Italy; mario.cinone@uniba.it (M.C.); luca.lacitignola@uniba.it (L.L.); roberta.rossi@uniba.it (R.R.); antoniociro.guaricci@uniba.it (A.C.G.); leonardo.resta@uniba.it (L.R.); maria.albrizio@uniba.it (M.A.); 2 Safari Zoo, 72015 Fasano, Italy; laris@libero.it

**Keywords:** oviduct, fallopian tubes, primates, glycohistochemistry, immunohistochemistry, scanning electron microscopy

## Abstract

**Simple Summary:**

The early crosstalk between the ovulated oocytes and the oviduct occurs in the fimbria, which is sensitive to the sex hormone fluctuation. Morphological and molecular changes were investigated in the oviductal fimbria epithelium of the baboon (*Papio hamadryas*) during the menstrual cycle. A well-differentiated epithelium consisting of ciliated and nonciliated cells was present only during the preovulatory phase when the epithelial surface displayed acidic glycans, complex fucosylated N-glycans, and oligolactosamine residues. Nonciliated cells contained small apical protrusions and thin microvilli, suggesting secretory and absorptive activities. In addition, nonciliated cells expressed HSP60 and HSP90 in the apical blebs as well as nuclear estrogen and progesterone receptors. Interestingly, ciliated cells displayed HSP70, HSP90, and progesterone receptors in the cilia. The findings, in addition to adding to knowledge of the baboon oviduct, may represent a benchmark for translational studies of the primate oviduct, including humans.

**Abstract:**

The oviductal fimbria is the first extraovarian anatomical structure that the cumulus–oocyte complex (COC) encounters, and is sensitive to sex hormone changes. The morphology, glycan pattern, expression of heat shock proteins (HSPs), estradiol receptor (ER), and progesterone receptor (PR) were investigated in the oviductal fimbria epithelium of the baboon (*Papio hamadryas*) during the menstrual cycle. The morphology was investigated by light and scanning electron microscopy; the glycopattern was characterized using conventional and lectin histochemistry; HSPs (60, −70, −90), ER, and PR were localized immunohistochemically. Well-differentiated ciliated and nonciliated cells were present only during the preovulatory phase. The nonciliated cells contained small apical protrusions and thin microvilli. During the preovulatory phase (1) the luminal surface of the fimbria displayed acidic glycans, complex N-glycans containing fucose, and oligolactosamine residues; (2) nonciliated cells expressed HSP60 and HSP90 in the apical blebs, HSP70 in the nucleus and cytoplasm, as well as nuclear ERα and PR; (3) ciliated cells showed HSP70 in the nucleus, cytoplasm, and cilia that also expressed HSP90 and PR. These results are related to the function of the fimbria where the early COC–oviduct crosstalk occurs and may represent a benchmark for translational studies of other primates.

## 1. Introduction

Oviducts (fallopian tubes in primates) are paired tubular organs and consist of five functionally unique segments: the fimbria, the infundibulum, the ampulla, the isthmus, and the uterotubal junction [1]. The fimbria, a fringe of finger-like mucosal folds projecting from the infundibulum, is proximal to the ovary and picks up the ovulated cumulus–oocyte complex (COC) and transports it to the next oviductal segment [1,2]. Therefore, this oviductal segment plays an important role in the reproductive process.

As another oviductal segment, the fimbria epithelium consists of ciliated and nonciliated (secretory) cells. Many studies in several species report that the morphology of fimbrial nonciliated and ciliated cells is very sensitive to changes in steroid hormone levels [3,4]. Estradiol induces hypertrophy, hyperplasia, and the differentiation of ciliated and secretory cells. In contrast, progesterone, in the presence or absence of estradiol, causes atrophy, deciliation, and loss of secretory activity within the mammalian oviduct [4].

Glycoproteins are complex molecules secreted from the mammalian oviduct. In the baboon *Papio anubis* oviduct, acidic protein dominates in the fimbrial region [5]. Fimbria glycoproteins could have a role in the early crosstalk between the ovulated oocytes and the oviduct in the transport of eggs to the infundibulum. The presence of glycoproteins in the fimbria epithelium has also been found in bovine [3] and rat [6] oviducts.

Heat shock proteins (HSPs) are among evolution’s most conserved protein families [7]. Some HSPs are constitutively expressed and participate in complex processes in living cells including cytoplasmic protein synthesis, transport, modification, and secretion [7,8], and are differently expressed during cell differentiation [9]. Their molecular size drives the classification of these proteins into families, and HSP60, 70, and 90, are the most studied. HSP60 exhibits a primary role in post-translational modifications of new proteins, repairing damaged ones and removing unnecessary peptides. This protein is mainly contained in the mitochondrial matrix [10]. The HSP70 family promotes the correct folding of newly synthesized proteins and modulates homeostasis. Under stress conditions, it takes action in cellular survival and tolerance [11]. Lastly, the HSP90 family has a role in maintaining protein conformation by associating with several intracellular proteins such as some receptors, calmodulin, actin, tubulin, and several kinases [12,13]. Expression of HSPs in the female reproductive system is sex hormone dependent. Studies on the expression of HSPs have been carried out in several oviductal segments of rats [14], cows [15], women [16], and baboons [17]. To our knowledge, no reports have been issued on the presence and localization of HSPs in the oviductal fimbriae.

Sex hormones exert their action on target genes by binding to intracellular receptors. The immunolocalization of estrogen receptors (ERs) and progesterone receptors (PRs) in primate oviducts has been carried out in women during the menstrual cycle [4,18,19,20,21]. As for non-human primates, only the expression of ERs has been investigated in the oviduct of cynomolgus macaques *(Macaca fascicularis*) [22]. All these above-cited studies report a nuclear immunolocalization of both ERs and PRs. However, PR immunoreactivity has also been found in the cilia of the human oviductal epithelium [21].

In addition to the transport of the ovulated COC in the infundibulum, the fimbria possesses the highest organoid-forming capacity compared to other anatomical regions of the oviducts [23,24,25]. Also, the tubal fimbria is involved in several pathological conditions such as adenocarcinoma [26,27], congenital ampullary atresia [28], and ovarian cancer [25].

Given the role of the oviductal fimbria in the reproductive process and its implications in pathological processes, the present study dealt with the changes in the morphology and the expression of glycans, HSPs, ERs, and PRs during the menstrual cycle of a non-human primate such as the baboon *Papio hamadryas*. Baboons can be considered a valuable model for translational medicine, being close to humans in their reproductive tracts and menstrual cycle [29].

## 2. Materials and Methods

### 2.1. Animals

In this study, fimbriae from the Fallopian tubes of fourteen healthy adult females of *Papio hamadryas* were used. The oviduct was removed by laparoscopic salpingectomy from nonpregnant females without endometrial damage, as revealed by abdominal ultrasonography.

The research was conducted during a birth control of *Papio hamadryas* housed in the Safari Zoo (Fasano (BR), South Italy). The clinical project was required and authorized with written informed consent by the zoo’s property (Leo 3000 S.p.a, c/o Safari Zoo) and approved by the Ethical Committee of the Department of Emergency and Organs Transplantation of the University of Bari-Aldo Moro (approval number: 05/2020). All surgical interventions were conducted following Italian law with respect to animal welfare.

### 2.2. Tissue Preparation

The identification of the menstrual cycle phases was described in a previous study [30].

**Figure 1:**
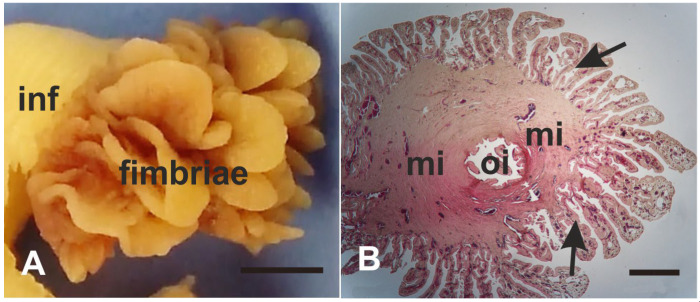
Macroscopic (**A**) and histological view (**B**) of baboon *Papio hamadryas* oviductal fimbriae stained with Hematoxylin-Eosin. In B, note the opening of the infundibulum (oi). oi, ostium of the infundibulum; mi, muscular of the infundibulum; arrow, mucosal folds of the fimbriae. Scale bar: (**A**) 2 mm; (**B**) 500 µm.

Oviducts from the follicular (n = 5), preovulatory (n = 4), and luteal (n = 5) phases of the baboons *Papio hamadryas* immediately after salpingectomy were immersed in 4% (*w*/*v*) phosphate-buffered (PBS) paraformaldehyde and fixed for 24 h at room temperature (RT). Then, the fimbria segment was separated from the adjacent segment of each oviduct (Figure 1) and processed for light microscopy and scanning electron microscopy (SEM) investigations.

The tissues destined for the light microscopy investigations were dehydrated in an ethanol series, cleared in xylene, and embedded in paraffin. Serial sections (5 μm thick) were cut, and, after de-waxing with xylene and hydration in an ethanol series of descending concentrations, were stained with Hematoxylin-Eosin for morphological analysis and with histochemical and immunohistochemical procedures to investigate the glycan pattern and the expression of HSPs, ER and PR.

For scanning electron microscopy (SEM) observations, dehydrated specimens were critical point dried using CO_2_, and, after mounting on stubs, were coated with gold-palladium in a sputter coater, and examined using an FEI QUANTA 250 (FEI Company, Milano, Italy).

The area occupied by the cilia was evaluated using 10 SEM microscopy fields randomly captured at 2500 magnification from the fimbria of each preovulatory subject, and analyzed using the image-analyzing program NIS Elements BR (Ver. 4.20) (Nikon, Japan). The area of each microscopic field measured 3600 μm^2^. Data are given as means ± standard deviation (SD).

No statistical analysis was applicable because the two cell types were not well differentiated and distinguishable in the other phases of the menstrual cycle.

### 2.3. Conventional Histochemistry

Sections were treated with (1) the periodic acid–Schiff (PAS) reaction for neutral glycans; (2) Alcian Blue pH 2.5 (AB 2.5)/PAS sequence to reveal combinations of acidic and neutral mucins; and (3) combined high iron diamine/AB 2.5 (HID/AB 2.5) for simultaneous staining of sulfated and non-sulfated acidic glycans [31].

### 2.4. Lectin Histochemistry

To characterize the glycan pattern, tissue sections were treated according to Desantis et al. (2022) [30]. Briefly, after rinsing in 0.05 M Tris-HCl-buffered saline (TBS), pH 7.4, the sections were incubated at RT for 1 h in the dark with appropriate dilutions of four fluorescent lectins (Table 1) diluted in the TBS.

All lectins were obtained from Vector Laboratories (Burlingame, CA, USA). After three rinses in TBS, slides were mounted in Vectashield Antifade Mounting Medium with DAPI (Vector Lab., Burlingame, CA, USA). Each experiment was repeated twice for each sample. Controls for lectin staining included (1) substitution of the substrate medium with buffer without lectin and (2) incubation with each lectin in the presence of its hapten sugar. All control experiments gave negative results.

### 2.5. Immunolocalization of HSPs, ERs, and PRs

Sections used to immunolocalize the HSPs, ERs, and PRs were de-waxed, re-hydrated and incubated for 15 min in methanol containing 3% H_2_O_2_ to inhibit endogenous peroxidase activity. After washing in PBS, non-specific binding sites were blocked by incubation in normal horse serum (NHS) (Vector Laboratories, Burlingame, CA, USA) for 30 min at RT and incubated overnight at 4 °C in a moist chamber with primary antibodies. The specification and dilution of the used primary antibodies are summarized in Table 2.

After the incubation with the primary antibody, the sections were washed with PBS for 15 min and the biotinylated universal antibody (Vector Laboratories, Burlingame, CA, USA) was added for 30 min at RT, followed, after a new 15 min PBS wash, by 30 min incubation with Vectastain Elite ABC Reagent (Vector Laboratories, Burlingame, CA, USA). After washing in PBS, staining was visualized by incubating the sections in DAB solution (Vector Laboratories, Burlingame, CA, USA). Then, the sections were dehydrated and mounted. The following negative controls were performed: (1) replacement of the primary antibodies with NHS in PBS or normal mouse serum, and (2) hybridization with a solution containing the primary antibody and a molar excess of its corresponding blocking peptide (sc-13115P for HSP60; sc-7298P for HSP70; sc515081PE for HSP90; sc-8002P for ERα). Under these conditions, staining was abolished. In addition, because the control peptide for PR was not available, a theoretical identity between the human (accession number DQ234979) and the *Papio anubis* (accession number DQ234986) gene sequences was checked, aligning the two sequences using the BLAST software from NCBI. The resulting identity was 96.88%.

## 3. Results

### 3.1. Morphology

**Figure 2:**
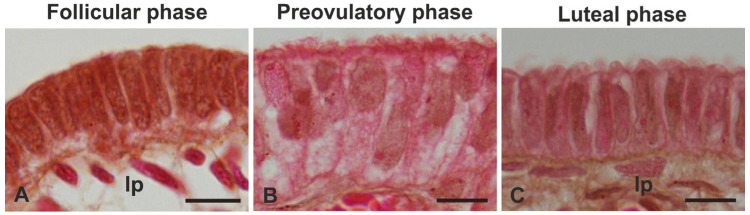
Light micrographs showing the morphological changes in the epithelium of baboon *Papio hamadryas* oviductal fimbriae during the menstrual cycle. Hematoxylin-Eosin staining. Scale bar: 10 µm.

The histomorphological analysis demonstrated that the epithelium lining the mucosa of the fimbriae reached the maximum height and differentiation status during the preovulatory phase (Figure 2) (see citation number 30 for morphometric details).

**Figure 3:**
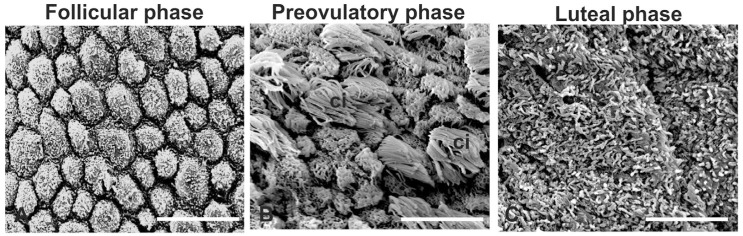
Scanning electron micrographs of the epithelium of baboon *Papio hamadryas* oviductal fimbriae during the menstrual cycle. ci, cilia. Scale bar: 8 µm.

SEM analysis (Figure 3) revealed well-differentiated ciliated and nonciliated cells only during the preovulatory phase. The nonciliated cells showed short microvilli on the apical membrane.

Since the cilia are essential for facilitating luminal fluid movement, oocyte pickup, and transport in the fimbria oviduct [2], the percentage area occupied by the cilia was evaluated and was 42.5 ± 19.4. Interestingly, the distribution of ciliated cells was not homogeneous along each fimbria, and the area ranged from 35% to 50%. During the luteal phase, the apical surface of the fimbria epithelium was characterized by cilia residues, whereas during the follicular phase, the absence of cilia was a prominent feature.

### 3.2. Glycohistochemistry

**Figure 4:**
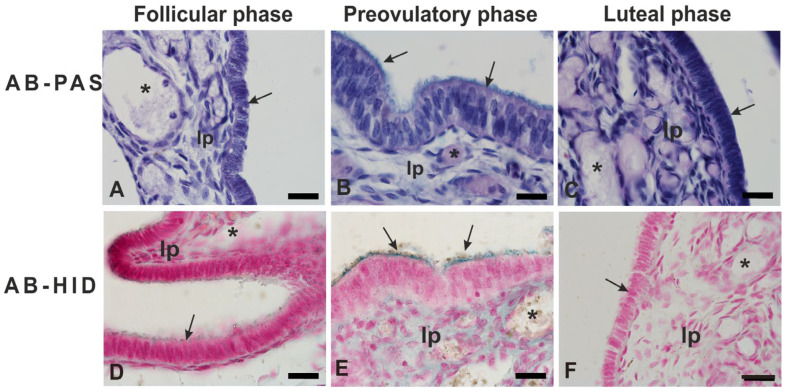
Conventional histochemical staining of fimbria epithelium of baboon *Papio hamadryas* oviduct during the menstrual cycle. Note the absence of PAS staining (**A**–**C**) and the presence of AB 2.5 staining (azur staining) during the follicular and preovulatory phase (**D**,**E**). HID staining (brown staining) was detected only during the preovulatory phase (**E**). In (**A**–**C**), nuclei were stained with Hematoxylin. In (**D**–**F**), nuclei were stained with nuclear fast red. lp, lamina propria; arrow, luminal surface of the epithelium; asterisk, blood vessel. Scale bar: 20 µm.

**Figure 5:**
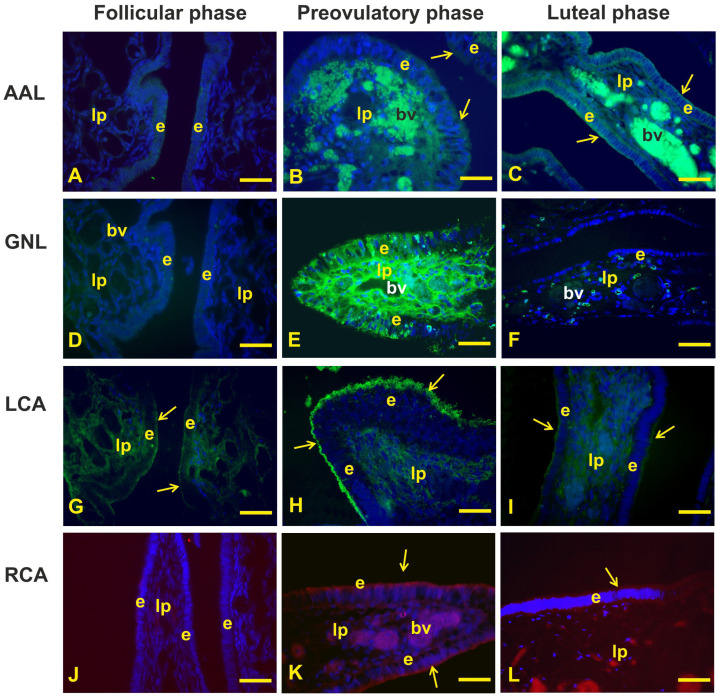
AAL, GNL, LCA, and RCA_120_ binding sites in the mucosal epithelium of baboon *Papio hamadryas* oviductal fimbriae during the menstrual cycle. (**A**–**C**), expression of aL-Fuc terminating glycans revealed with LTA. (**D**–**F**), presence of terminal α1-3mannose residue detected by GNL. (**G**–**I**), localization of complex N-linked glycans by means of LCA. (**J**–**L**), lactosamine terminating glycans identified by RCA_120_. Note that the investigated glycans were mainly expressed during the preovulatory phase compared to other menstrual cycle phases. bv, blood vessel; e, epithelium; lp, lamina propria; arrow, luminal surface of the epithelium. Scale bar: 40 µm. AAL, GNL, and LCA were FITC-conjugated lectins. RCA was TRITC-conjugated lectin.

The results of conventional and lectin staining patterns of the fimbria epithelium are summarized in Table 3 and Figure 4 and Figure 5.

PAS did not stain the epithelium during the entire menstrual cycle (Figure 4A–C), while AB2.5 stained very weakly the apical surface of the follicular fimbriae (Figure 4A,D) and strongly the apical surface of the preovulatory fimbriae (blue staining) (Figure 4B,E). HID gave barely visible staining (brown staining) only on the apical surface of the fimbriae during the preovulatory phase (Figure 4E). The fimbriae of the luteal phase gave a negative reaction with PAS, AB2.5, and HID (Figure 4C,F).

The fucosylated glycans (AAL reactivity) were absent during the follicular phase (Figure 5A), whereas they were weakly expressed in the apical cytoplasm of the fimbria epithelium during the preovulatory phase (Figure 5B) and barely visible in the apical surface of fimbriae during the luteal phase (Figure 5C).

The lectin GNL, specific for terminal α1-3mannose, showed a moderate and scattered granular reactivity in the cytoplasm of some epithelial cells during the preovulatory phase, while during the follicular and luteal phases did not find binding sites (Figure 5D–F).

The complex N-linked glycans investigated with LCA were strongly expressed in the apical surface of the fimbriae during the preovulatory phase, whereas they were faintly visible in the apical surface of the follicular and luteal phase fimbriae (Figure 5G–I).

Oligosaccharides terminating with lactosamine (RCA_120_ affinity) were not found in the fimbrial epithelium during the follicular phase (Figure 5J). On the other hand, they were well visible in the apical surface of the fimbria during the preovulatory phase (Figure 5K) and barely visible during the luteal phase (Figure 5L).

### 3.3. Immunolocalization of HSPs

**Figure 6:**
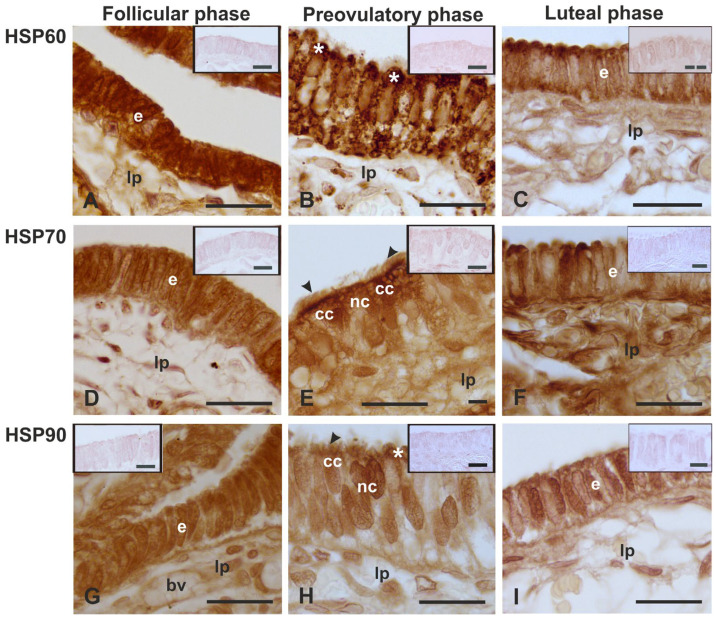
Immunostaining pattern of the HSP60 (**A**–**C**), 70 (**D**–**F**), 90 (**G**–**I**) in the mucosal epithelium of baboon *Papio hamadryas* oviductal fimbriae during the menstrual cycle. The inset images display the absence of immunoreactivity in negative controls. cc, ciliated cell; e, epithelium; lp, lamina propria; nc, nonciliated cell; arrowhead, cilia; *, apical bleb. Scale bar: 20 µm; insets, 10 µm.

A variable expression pattern of HSP60, 70, and 90 was observed in the epithelium of the fimbria during the menstrual cycle (Table 4 and Figure 6).

HSP60 immunoreactivity was strongly observed in the cytoplasm and nucleus of the epithelial cells during the follicular phase (Figure 6A). The HSP60 immunostaining decreased in the other phases of the menstrual cycle. This protein showed a granular staining pattern and was present from the basal cytoplasm to the apical cytoplasm of nonciliated cells during the preovulatory phase (Figure 6B). In the luteal phase, the fimbria epithelium displayed both negative and positive HSP60-immunoreactive cells. The latter contained also a positive nucleus (Figure 6C).

HSP70 was detected in the cytoplasm and nucleus of all cells constituting the fimbria epithelium during the follicular phase (Figure 6D) and preovulatory phase, which displayed chaperonin also in the cilia (Figure 6E). During the luteal phase, the HSP70 immunoreactive pattern (Figure 6F) was similar to that described for HSP60.

HSP90 immunoreactivity was found in the cytoplasm and nucleus of cells constituting the fimbria epithelium during the follicular phase (Figure 6G) and preovulatory phase (Figure 6H). In this phase, the immunostaining was characteristically observed in the apical blebs of the nonciliated cells and in the cilia. The HSP90 immunolocalization pattern of the luteal phase fimbria epithelium (Figure 6I) appeared similar to the HSP60 and HSP70 ones.

### 3.4. Immunolocalization of ER and PR

**Figure 7:**
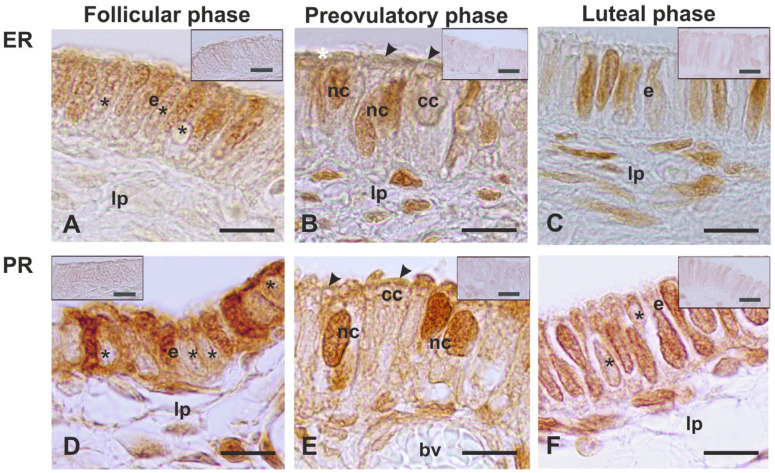
ERα and PR immunostaining pattern in the baboon *Papio hamadryas* oviductal fimbriae during the menstrual cycle. (**A**–**C**), ERα was present only in the nuclei. (**D**–**F**), PR immunoreactivity was observed unevenly in the cytoplasm and nucleus of epithelial cells during the follicular and luteal phases, whereas it was present in the nuclei of the nonciliated cells and in the cilia during the preovulatory phase. The inset images display the absence of immunoreactivity in negative controls. cc, ciliated cell; e, epithelium; lp, lamina propria; nc, nonciliated cell; arrowhead, cilia; asterisk, negative nucleus. Scale bar: 10 µm.

The fimbria epithelium displayed a different ERα and PR distribution pattern during the menstrual cycle (Table 5 and Figure 7).

ERα was not found in all nuclei of the fimbrial epithelium during the follicular and luteal phases (Figure 7A,C), or in the nuclei of the nonciliated cells during the preovulatory phase (Figure 7B). The staining intensity for ERα did not change significantly during the menstrual cycle.

PR immunostaining was stronger than the ERα one. During the follicular phase, the PR was detected in the nucleus and cytoplasm of epithelial cells, alternating with cells displaying a weakly stained cytoplasm (Figure 7D). The preovulatory fimbria epithelium displayed strong PR-immunoreactivity in the nucleus of nonciliated cells and at the base of the ciliary stalk (Figure 7E). The luteal-phase fimbria epithelium showed the PR in the nucleus of most cells and a few cells’ cytoplasm (Figure 7F).

## 4. Discussion

The oviductal fimbriae are the first anatomical structures with which COC interacts outside the ovary and have an essential role in picking up the ovulated COC and transporting it to the infundibulum. Therefore, the knowledge of fimbria’s structural and molecular characteristics can help to understand the events involved in the complex phenomenon of COC uptake and gateway into the oviduct. This study reports, for the first time, the changes occurring in the epithelium lining the oviductal fimbria of a non-human primate, specifically the baboon *Papio hamadryas*, during the menstrual cycle as observed by light and scanning electron microscopy, glycan histochemistry, and immunolocalization of HSP-60, -70, -90, ERs, and PRs.

### 4.1. Morphology

The morphological observations carried out by light microscopy and SEM investigations revealed that the oviductal fimbriae of the baboon *Papio hamadryas* displayed a well-differentiated single-layered columnar epithelium constituted of ciliated and nonciliated cells only during the preovulatory phase of the menstrual cycle when the height of the epithelium was higher compared to the follicular and luteal phases and the two types of cells were not distinguishable. These morphological features have been related to the blood levels of the sex hormones [30] that, as is well known, regulate the gene expression of the oviductal epithelial cells.

In the present study, the percentage of both ciliated and nonciliated cells was evaluated because we used 5 µm thick sections for the histological observations. This thickness does not guarantee a reliable count of both cell types, unlike the semi-thin sections (1.5 µm thick sections) used in the study of the oviduct of other primates [32,33]. Therefore, we used SEM micrographs taken in the fimbriae of the preovulatory baboons. Surprisingly, we observed an uneven distribution of ciliated cells along the fimbriae epithelia. Cilia can hide the surface of adjacent nonciliated cells. Thus, in preovulatory animals, the surface area occupied by cilia was calculated to evaluate the extent of action of the ciliated cells. The results revealed that the surface area occupied by cilia ranged from 35% to 50%. This finding suggests that the mucosal folds of the fimbriae consist of areas playing different roles. In primates, the COC is ovulated into the peritoneal cavity and the cilia of the fimbriae create a current of peritoneal fluid toward the ostium of the infundibulum, facilitating oocyte passage into this segment of the oviduct [2].

SEM observations also displayed that nonciliated cells of the preovulatory phase contained small apical protrusions covered by thin microvilli. This finding suggests that the oviductal fimbriae of baboons *Papio hamadryas* are involved in the absorption of the luminal fluid. However, secretory activity cannot be ruled out since TEM investigations revealed the presence of both microvilli and secretory granules in the nonciliated cells of fimbriae from baboon *Papio cynocephalus* oviducts [32].

### 4.2. Glycohistochemistry

The conventional histochemistry (AB2.5, and HID/AB2.5) as well as lectin histochemistry revealed a strong presence of glycans in the fimbria epithelium of the preovulatory phase compared to the other phases of the menstrual cycle. The apical surface of the preovulatory-phase epithelium showed the presence of non-sulfated and, to a lesser extent, sulfated acidic glycans, revealed with AB 2.5 and HID, respectively. A dominant presence of the acidic glycoprotein was also detected in the fimbria region at the midcycle and in intact estradiol-treated baboons *Papio Anubis* [5]. The high presence of acidic glycans on the epithelial surface highlights the presence of negatively charged molecules that could prevent the adhesion of the COC to the epithelium and promote its flow into the infundibulum. In the present study, the PAS staining gave negative results. A similar finding has been observed in the fimbria of rat oviducts [6]. No report exists on the PAS affinity of the fimbrial epithelium of primate oviducts.

The study of the glycan profile in the epithelium of the baboon *Papio hamadryas* fimbrial oviduct using lectin histochemistry has already been performed [30]. Due to the high importance of the glycoproteins in reproductive events, we further investigated the glycoprofile of the fimbria epithelium during the menstrual cycle by detection of additional fucosylated and N-linked glycans, using the lectins AAL, GNL, LCA, and RCA_120_. Fucosylated carbohydrate moieties are involved in a variety of physiological and pathological processes [34] including reproduction [35]. The N-linked glycans are involved in the structure, activity, and antigenicity of proteins [36]. AAL revealed the presence of fucosylated glycans in the apical cytoplasm during the preovulatory phase, which was reduced during the luteal phase. These findings indicate that the fimbrial epithelium produces α1,6- and α1,4-fucosylated glycans that had hitherto not been found.

As for the N-linked glycans, the epithelial surface displayed the highest presence of N-glycans that were core-fucosylated (LCA positivity) [37] during the preovulatory phase when the entire cytoplasm of scattered cells contained terminal mannose in monoantenary and bi/triantenary oligosaccharides (GNL reactivity) [37]. In addition, RCA_120_, which recognizes oligolactosamine chains in N-linked glycans [38], showed a binding pattern like LCA. This finding should not be surprising since RCA_120_ can also bind oligolactosamine chains containing fucose residues [38]. All these findings indicate that the glycoprofile of the fimbrial epithelium is regulated by the sex hormone fluctuations occurring during the menstrual cycle. As in other mammals [3], the primate oviduct (namely baboon *Papio anubis*) also secretes a family of estrogen-dependent glycoproteins that appear to be present only in mature secretory cells [39]. Although the role of the highly complex glycopattern observed during the preovulatory phase is not known, it could be potentially related to the maximum degree of differentiation and function of the epithelium during this phase of the menstrual cycle when the ovulated COC is picked up and the early cross-talk between the ovulated oocytes and the oviduct occurs. Furthermore, it cannot be ruled out that the glycoproteins observed on the luminal surface of the epithelium are molecules secreted by nonciliated cells. It has been reported that fimbrial-secreted glycoproteins could bind to the cumulus matrix, zona pellucida, and perivitelline space of mouse newly ovulated oocytes [40].

### 4.3. Immunolocalization of the HSPs

To the best of our knowledge, no study concerning the presence of HSPs in the fimbriae of mammalian oviducts has been previously published. The immunohistochemistry revealed changes in the localization and expression of the investigated HSPs in the fimbria epithelium during the phases of the menstrual cycle.

HSP60 was found in the nucleus and cytoplasm of the epithelial cells during the follicular and luteal phases. During the preovulatory phase, when the nonciliated and ciliated cells were well distinguishable, HSP60 immunostaining appeared only in the nonciliated cells that showed a granular pattern in the cytoplasm and were evenly concentrated in the apical blebs. The observed granular pattern agrees with the mitochondrial and cytoplasm localization of this chaperonin, as reported in previous studies [8,10]. It has been demonstrated that HSP60 is involved in protein folding and assemblage of oligomeric protein complexes and in eliminating misfolded proteins by targeting them to cellular proteolytic machinery [10]. A similar fimbria HSP60 staining pattern has been observed in the nonciliated cells of baboon *Papio hamadryas* oviductal ampulla during the preovulatory phase [17]. The presence of HSP60 in the apical blebs of nonciliated cells could be a sign of its secretion from cells, suggesting the role of this chaperonin in the extracellular system. Thus, it is possible to hypothesize the role of HSP60 in regulating the chemical environment that COC meets during the flow in the baboon oviduct. HSP60 has been detected in the human follicular fluid [7]. The secretion of HSP60 by epithelial cells has also been reported in non-reproductive anatomical districts such as bronchial epithelial cells [41].

The chaperonin HSP70 was detected in the cytoplasm and the nucleus of epithelial cells during the entire menstrual cycle. These results are in line with previous reports on the HSP70 cellular localization, such as in the cytosol, nucleus, endoplasmic reticulum, and mitochondria [see 8 for references]. This protein keeps the dynamic balance of the synthesis, folding, degradation, and translocation of proteins [8]. The cytoplasmic and nuclear localization of HSP70 may be related to the protective role of this protein against DNA damage [42]. During the preovulatory phase, HSP70 was found also in the cilia. The HSP70 in the cilia has also been observed in the baboon *Papio hamadryas* ampulla [17]. In cilia, the HSP70 chaperone may assist in targeting tubulin as well as participate in the assembly of the axoneme [43]. HSP70 has been found in the cilia of rabbit trachea [43] and cat ductuli efferentes [42].

The localization of HSP90 was found in the cytoplasm of the epithelium during the follicular and luteal phases, and in the nuclei throughout the menstrual cycle. During the preovulatory phase, HSP90 was detected in the apical blebs of nonciliated cells and cilia of ciliated cells. This finding indicates that secretion of HSP90 could occur in the fimbria oviduct. HSP90 has also been immunohistochemically detected in the apical secretory blebs of the epididymis and vas deferens of cats [42]. It has been suggested that the ciliary HSP90 is involved in the stability of axonemal tubulin and the regulation of ciliary beating [13]. Therefore, the current study findings suggest that HSP90 could contribute to the chemical composition of the fimbria surface and the moving of new ovulated COC towards the infundibulum. Recently the expression of HSP90 in the apical region of nonciliated cells and cilia of the oviductal ampulla in baboon *Papio hamadryas* during the preovulatory phase has been detected [17]. It has been reported that HSP90 constitutes oviductal secretions [44] and could regulate estrogen receptor signaling [45]. Several studies support the key role of estrogen in the expression of HSPs [46,47].

### 4.4. Immunolocalization of ERs and PRs

The fimbria displayed ERα immunoreactivity in the nuclei of the epithelial cells throughout the menstrual cycle. The nuclear ERα was not detected in all cells during the follicular and luteal phases; on the contrary, it was found in the nuclei of the epithelial nonciliated cells of the preovulatory-phase fimbriae. These findings suggest that the nuclear ERα positivity observed during the follicular and luteal phases could belong to undifferentiated and dedifferentiated nonciliated cells, respectively. Studies on oviductal ER immunolocalization in the non-human primate are scanty. ER has been immunolocalized in the nuclei of the fimbriae of estradiol-treated cynomolgus macaques *(Macaca fascicularis)* [22]. In addition, Erα has been immunolocalized to the nuclei of the epithelial cells of the human oviductal fimbriae during all phases of the menstrual cycle [19]. The present study did not display a change in the staining intensity of ERα during the menstrual cycle. This finding is consistent with studies conducted in the human fallopian tube, which, unlike the endometrial epithelium, displayed a constant expression of Erα across the menstrual cycle [19,20]. Since oviductal Erα is not downregulated when the tissue is exposed to peak levels of circulating progesterone, it has been postulated that the fallopian tube epithelium may be more responsive to androgens and less responsive to estradiol and progesterone [19,20]. None the above-cited reports specified which cell type contained the nuclear ER. In rat oviducts, the ERα was found only in the nuclei of the nonciliated cells, which showed weak changes in the staining intensity during the estrous cycle [48]. It has been reported that ER signaling is not essential for ciliated epithelial cell differentiation because the presence of cilia was detected in the oviduct of ERα-deficient mice [49]. 

As for the PR, we found this receptor in the nucleus and the cytoplasm of the epithelial cells in fimbriae from follicular- and luteal-phase baboons. Interestingly, the PR was found in the nucleus of nonciliated cells and to a minor extent in the cilia during the preovulatory phase. PR immunoreactivity has also been observed in the nucleus of nonciliated cells and the lower region of the cilia in mouse and human oviductal epithelia [21].

In the current study, no significant change in the staining intensity of PR was detected. This finding is consistent with reports on the human fallopian tube in which a static expression of PR has been observed in the oviductal epithelium across the menstrual cycle [19,20].

Regarding the presence of PR in the cilia, it has been suggested that PRs could play a role in the regulation of oviductal ciliary activity, including the pickup and transport of the COC via extracellular signals [21,50]. It has been reported that the progesterone present in the follicular fluid at ovulation, as well as secreted by COC, could produce a signaling pathway involved in the activation of specific Ca^++^-selective ion channels that could regulate the functions of ciliated cells [21,51].

## 5. Conclusions

This study demonstrated that the nonciliated and ciliated cells are well differentiated in the fimbrial epithelium during the preovulatory phase of the menstrual cycle of the baboon *Papio hamadryas*. Their luminal surface glycans could regulate the crosstalk between the COCs and promote ciliary beating by allowing the flow of COC into the infundibulum. The nonciliated cells are also implicated in the absorption of the luminal fluid and in the secretion of glycans, HSP60, and HSP90, thus modifying the chemical composition of the lumen content. The expression of HSP70 and HSP90 in the cilia of the ciliated cells suggests a positive role of these chaperonins in their movement. The absence of ERα in the ciliated cells suggests that ER signaling could be not essential for the differentiation of the ciliated cells. In contrast, PRs in the cilia could activate extracellular signals for the ciliary beat. These results provide new data on the first anatomical structure, such as the oviductal fimbria of a primate phylogenetically close to humans, with which the ovulated COC interacts.

## Figures and Tables

**Table 1:** Lectin used, their sugar specificities, and the inhibitory sugars used in control experiments.

Lectin Abbreviation	Source of Lectin	µg/mL	Sugar Specificity	Inhibitory Sugar
AAL	*Aleuria aurantia*	25	Fucα1-3/1-4/1-2/l-6	Fucose
GNL	*Galanthusnivalis*	25	Terminal α1-3Man	αMet-Mannose
LCA	*Lens culinaris*	25	Man1-2Man1-3Man	αMet-Mannose
RCA_120_ *	*Ricinus communis*	25	Galβ1-4GlcNAcβ	Galactose

Fuc, fucose; Gal, galactose; GlcNAc, N-acetylglucosamine; Man, mannose; Met, methyl. Non-marked lectins were FITC (fluorescein isothiocyanate)-labelled lectins. *, TRITC (rhodamine)-labeled lectin.

**Table 2:** The primary antibodies used for immunohistochemical detection of ER, PR, HSP 60,70,90 in the fimbriae epithelia of baboon *Papio hamadryas* uterine tube during the menstrual cycle.

Primary Antibody	Type	Epitope	Dilution	Supplier	Catalog Number
HSP60 (H-1)	mouse monoclonal	Amino acids 28–62 near the N-terminus of human HSP60	1:200	Santa Cruz Biotechnology, Inc., Santa Cruz, CA, USA	sc-13115
HSP70 (B-6)	mouse monoclonal	Amino acids 580–601 at the C-terminus of human HSP 70	1:200	Santa Cruz Biotechnology, Inc., Santa Cruz, CA, USA	sc-7298
HSP90α (F-2)	mouse monoclonal	Amino acids 342–382 within an internal region of human HSP90α	1:200	Santa Cruz Biotechnology, Inc., Santa Cruz, CA, USA	sc-515081
ERα (F-10)	mouse monoclonal	Amino acids 570–595 at the C-terminus of ERα of human origin	1:50	Santa Cruz Biotechnology, Inc., Santa Cruz, CA, USA	sc-8002
PR (AB-52)	mouse monoclonal	native human PR-A and PR-B	1:50	Santa Cruz Biotechnology, Inc., Santa Cruz, CA, USA	sc-810

ER, estrogen receptor; HSP, heat shock protein; PR, progesterone receptor.

**Table 3:** Conventional and lectin histochemistry staining pattern of the epithelium lining the fimbriae of baboon *Papio hamadryas* oviduct during the menstrual cycle.

	Follicular Phase	Preovulatory Phase	Luteal Phase
PAS	−	−	−
AB 2.5	±	++as	−
HID	−	±as	−
AAL	−	+ac	±as
GNL	−	++	−
LCA	±as	+++as	+as
RCA_120_	−	++as	±as

AB 2.5, Alcian Blue pH 2.5; ac, apical cytoplasm; as, apical surface; HID, high iron diamine; PAS, periodic acid–Schiff. −, negative reaction; ±, faintly visible reaction; +, ++, +++, weak, moderate, strong reactions.

**Table 4:** Expression of the HSP60, -70, -90 in the mucosal epithelia of baboon *Papio hamadryas* oviductal fimbriae during the menstrual cycle.

	Follicular Phase	Preovulatory Phase		Luteal Phase
		Nonciliated cells	Ciliated cells	
HSP60	+++/+++n	+g/+++ab	−	+ */±n
HSP70	+/+n	++/+n	++/++ci/+n	+ */±n
HSP90	+/+n	±/+ab/+n	±/+ci	+ */±n

ab, apical bleb; ci, cilia; g, granular staining; n, nucleus; *, positivity was not in all cells. If not specified, the positivity was detected in nonciliated and ciliated cells. −, ±, +, ++, +++, negative, barely, weakly, moderately, strongly visible immunostaining.

**Table 5:** Expression of the Erα and PR in the mucosal epithelium of baboon *Papio hamadryas* oviductal fimbriae during the menstrual cycle.

	Follicular Phase	Preovulatory Phase		Luteal Phase
		Nonciliated cells	Ciliated cells	
ERα	+n *	+n	−	+n *
PR	++cn */+ *	++n	+ci	± */++n

ci, cilia; cn, cytoplasm and nucleus; n, nucleus; *, positivity was not in all cells. If not specified, the positivity was detected in nonciliated and ciliated cells. −, ±, +, ++, negative, barely, weakly, strongly visible immunostaining.

## Data Availability

The data presented in this study are available on request from the corresponding author.

## References

[1] Barton B.E., Herrera G.G., Anamthathmakula P., Rock J.K., Willie A.M., Harris E.A., Takemaru K.-I., Winuthayanon W. (2020). Roles of steroid hormones in oviductal function. Reproduction.

[2] Yuan S., Wang Z., Peng H., Ward S.M., Hennig G.W., Zheng H., Yan W. (2021). Oviductal motile cilia are essential for oocyte pickup but dispensable for sperm and embryo transport. Proc. Natl. Acad. Sci. USA.

[3] Abe H. (1996). The mammalian oviductal epithelium: Regional variations in cytological and functional aspects of the oviductal secretory cells. Histol. Histopathol..

[4] Mazur E.C., Large M.J., DeMayo F.J., Knobil E., Neill J., IV (2015). Human Oviduct and Endometrium: Changes over the Menstrual Cycle. The Physiology of Reproduction.

[5] Verhage H.G., Fazleabas A.T. (1988). The in vitro synthesis of estrogen-dependent proteins by the baboon (*Papio anubis*) oviduct. Endocrinology.

[6] Lee L.-H., Sugimura M., Kudo N. (1976). Segmentation of the rat oviduct. Jpn. J. Vet. Res..

[7] Neuer A., Spandorfer S.D., Giraldo P., Dieterle S., Rosenwaks Z., Witkin S.S. (2000). The role of heat shock proteins in reproduction. Hum. Reprod. Update.

[8] Hu C., Yang J., Qi Z., Wu H., Wang B., Zou F., Mei H., Liu J., Wang W., Liu Q. (2022). Heat shock proteins: Biological functions, pathological roles, and therapeutic opportunities. MedComm.

[9] Cinone M., Albrizio M., Guaricci A.C., Lacitignola L., Desantis S. (2024). Testicular expression of heat shock proteins 60, 70, and 90 in cryptorchid horses. Theriogenology.

[10] Malik J.A., Lone R. (2021). Heat shock proteins with an emphasis on HSP 60. Mol. Biol. Rep..

[11] Kopecek P., Altmannova K., Weigl E. (2001). Stress proteins: Nomenclature, division and functions. Biomed. Pap..

[12] Sima S., Richter K. (2018). Regulation of the Hsp90 system. Biochim. Biophys. Acta (BBA)-Mol. Cell Res..

[13] Takaki E., Fujimoto M., Nakahari T., Yonemura S., Miyata Y., Hayashida N., Yamamoto K., Vallee R.B., Mikuriya T., Sugahara K. (2007). Heat shock transcription factor 1 is required for maintenance of ciliary beating in mice. J. Biol. Chem..

[14] Mariani M.L., Souto M., Fanelli M.A., Ciocca D.R. (2000). Constitutive expression of heat shock proteins hsp25 and hsp70 in the rat oviduct during neonatal development, the oestrous cycle and early pregnancy. J. Reprod. Fertil..

[15] Boilard M., Reyes-Moreno C., Lachance C., Massicotte L., Bailey J.L., Sirard M.A., Leclerc P. (2004). Localization of the chaperone proteins GRP78 and HSP60 on the luminal surface of bovine oviduct epithelial cells and their association with spermatozoa. Biol. Reprod..

[16] Lachance C., Bailey J.L., Leclerc P. (2007). Expression of HSp60 and Grp78 in the human endometrium and oviduct, and their effect on sperm functions. Hum. Reprod..

[17] Albrizio M., Desantis S., Lacitignola L., Laricchiuta P., Guaricci A.C., Cinone M. (2024). The abundance and localization of heat shock proteins (HSP)-60, -70, and -90 in the oviductal ampulla of hamadryas baboon (*Papio hamadryas*) during the menstrual cycle. Vet. Res. Com..

[18] Amso N.N., Crow J., Shaw R.W. (1994). Comparative immunohistochemical study of oestrogen and progesterone receptors in the Fallopian tube and uterus at different stages of the menstrual cycle and the menopause. Hum. Reprod..

[19] Horne A.W., King A.E., Shaw E., McDonald S.E., Williams A.R.W., Saunders P.T., Critchley H.O.D. (2009). Attenuated sex steroid receptor expression in Fallopian tube of women with ectopic pregnancy. J. Clin. Endocrinol. Metab..

[20] Maclean A., Bunni E., Makrydima S., Withington A., Kamal A.M., Valentijn A.J., Hapangama D.K. (2020). Fallopian tube epithelial cells express androgen receptor and have a distinct hormonal responsiveness when compared with endometrial epithelium. Hum. Reprod..

[21] Teilmann S.C., Clement C.A., Thorup J., Byskov A.G., Christensen S.T. (2006). Expression and localization of the progesterone receptor in mouse and human reproductive organs. J. Endocrinol..

[22] McClellan M.C., West N.B., Tacha D.E., Greene G.L., Brenner R.M. (1984). Immunocytochemical localization of estrogen receptors in the macaque reproductive tract with monoclonal antiestrophilins. Endocrinology.

[23] Ghosh A., Syed S.M., Kumar M., Carpenter T.J., Teixeira J.M., Houairia N., Negi S., Tanwar P.S. (2020). In vivo cell fate tracing provides no evidence for mesenchymal to epithelial transition in adult fallopian tube and uterus. Cell Rep..

[24] Lawson E.F., Ghosh A., Blanch V., Grupen C.G., Aitken R.J., Lim R., Drury H.R., Baker M.A., Gibb Z., Tanwar P.S. (2023). Establishment and characterization of oviductal organoids from farm and companion animals. Biol. Reprod..

[25] Xie Y., Park E.S., Xiang D., Li Z. (2018). Long-term organoid culture reveals enrichment of organoid-forming epithelial cells in the fimbrial portion of mouse fallopian tube. Stem. Cell Res..

[26] Huang H.S., Chu S.C., Hsu C.F., Chen P.C., Ding D.C., Chang M.Y., Chu T.Y. (2015). Mutagenic, surviving and tumorigenic effects of follicular fluid in the context of p53 loss: Initiation of fimbria carcinogenesis. Carcinogenesis.

[27] Medeiros F., Muto M.G., Lee Y., Elvin J.A., Callahan M.J., Feltmate C., Garber J.E., Cramer D.W., Crum C.P. (2006). The tubal fimbria is a preferred site for early adenocarcinoma in women with familial ovarian cancer syndrome. Am. J. Surg. Pathol..

[28] Tallon N., Akbar G., Mccomb P. (2013). Congenital ampullary atresia of the fallopian tube and the coexistence of fimbrial tissue. J. Genit. Syst. Disord..

[29] Bauer C. (2015). The baboon (*Papio* sp.) as a model for female reproduction studies. Contraception.

[30] Desantis S., Albrizio M., Lacitignola L., Laricchiuta P., Cinone M. (2022). Modification of morphology and glycan pattern of the oviductal epithelium of baboon *Papio hamadryas* during the menstrual cycle. Animals.

[31] Spicer S.S. (1965). Diamine methods for differentiating mucosubstances histochemically. J. Histochem. Cytochem..

[32] Odor D.L., Augustine J.R. (1995). Morphological study of changes in the baboon oviductal epithelium during the menstrual cycle. Microsc. Res. Tech..

[33] Verhage H.G., Mavrogianis P.A., Boice M.A., Li W., Fazleabas A.T. (1990). Oviductal epithelium of the baboon: Hormonal control and the immuno-gold localization of oviduct-specific glycoproteins. Am. J. Anat..

[34] Li J., Hsu H.C., Mountz J.D., Allen J.G. (2018). Unmasking fucosylation: From cell adhesion to immune system regulation and diseases. Cell Chem. Biol..

[35] Domino S.E., Zhang L., Gillespie P.J., Saunders T.L., Lowe J.B. (2001). Deficiency of reproductive tract α(1,2)fucosylated glycans and normal fertility in mice with targeted deletions of the FUT1 or FUT2 α(1,2)fucosyltransferase locus. Mol. Cell. Biol..

[36] Stanley P., Taniguchi N., Aebi M., Varki A. (2017). N-glycans. Essentials of Glycobiology.

[37] Goumenou A., Delaunay N., Pichon V. (2021). Recent advances in lectin-based affinity sorbents for protein glycosylation studies. Front. Mol. Biosci..

[38] Chandrasekaran E.V., Xue J., Xia J., Khaja S.D., Piskorz C.F., Locke R.D., Neelamegham S., Matta K.L. (2016). Novel interactions of complex carbohydrates with peanut (PNA), *Ricinus communis* (RCA-I), *Sambucus nigra* (SNA-I) and wheat germ (WGA) agglutinins as revealed by the binding specificities of these lectins towards mucin core-2 O-linked and N-linked glycans and related structures. Glycoconj. J..

[39] Verhage H.G., Fazleabas A.T., Mavrogianis P.A., O’Day-Bowman M.B., Donnelly K.M., Arias E.B., Jaffe R.C. (1997). The baboon oviduct: Characteristics of an oestradiol-dependent oviduct-specific glycoprotein. Hum. Reprod. Update.

[40] Lyng R., Shur B.D. (2009). Mouse oviduct-specific glycoprotein is an egg-associated ZP3-independent sperm-adhesion ligand. J. Cell Sci..

[41] Sangiorgi C., Vallese D., Gnemmi I., Bucchieri F., Balbi B., Brun P., Leone A., Giordano A., de Macario E.C., Macario A.J. (2017). HSP60 activity on human bronchial epithelial cells. Int. J. Immunopathol. Pharmacol..

[42] Liman N. (2023). Heat shock proteins are differentially expressed in the domestic cat (*Felis catus*) testis, epididymis, and vas deferens. Microsc. Microanal..

[43] Stephens R.E., Lemieux N.A. (1999). Molecular chaperones in cilia and flagella: Implications for protein turnover. Cell Motil. Cytoskelet..

[44] Saint-Dizier M., Schoen J., Chen S., Banliat C., Mermillod P. (2020). Composing the early embryonic microenvironment: Physiology and regulation of oviductal secretions. Int. J. Mol. Sci..

[45] Suuronen T., Ojala J., Hyttinen J.M.T., Kaarniranta K., Thornell A., Kyrylenko S., Salminen A. (2008). Regulation of ERα signaling pathway in neuronal HN10 cells: Role of protein acetylation and Hsp90. Neurochem. Res..

[46] Olazabal U.E., Pfaff D.W., Mobbs C.V. (1992). Sex differences in the regulation of heat shock protein 70 kDa and 90 kDa in the rat ventromedial hypothalamus by estrogen. Brain Res..

[47] Sirotkin A.V., Bauer M. (2011). Heat shock proteins in porcine ovary: Synthesis, accumulation and regulation by stress and hormones. Cell Stress Chaperons.

[48] Okada A., Ohta Y., Inoue S., Hiroi H., Muramatsu M., Iguchi T. (2003). Expression of estrogen, progesterone and androgen receptors in the oviduct of developing, cycling and pre-implantation rats. J. Mol. Endocrinol..

[49] Okada A., Ohta Y., Brody S.L., Watanabe H., Krust A., Chambon P., Iguchi T. (2004). Role of foxj1 and estrogen receptor alpha in ciliated epithelial cell differentiation of the neonatal oviduct. J. Mol. Endocrinol..

[50] Wessel T., Schuchter U., Walt H. (2004). Ciliary motility in bovine oviducts for sensing rapid non-genomic reactions upon exposure to progesterone. Horm. Metab. Res..

[51] Hunter M.I., Thies K.M., Winuthayanon W. (2024). Hormonal regulation of cilia in the female reproductive tract. Curr. Opin. Endocr. Metab. Res..

